# Patients with chronic kidney disease stage 3b: do general practitioners and nephrologists follow KDIGO guidelines?

**DOI:** 10.1590/2175-8239-JBN-2025-0229en

**Published:** 2026-04-17

**Authors:** Juliana Gazzi Macedo, Alessandra Maciel Almeida, Helady Sanders-Pinheiro, Raquel Aparecida Fabreti-Oliveira

**Affiliations:** 1 Faculdade de Ciências Médicas de Minas Gerais, Belo Horizonte, MG, Brazil.; 2 Universidade Federal de Minas Gerais, Belo Horizonte, MG, Brazil.; 3 Universidade Federal de Juiz de Fora, Hospital Universitário, Unidade de Transplante Renal, Juiz de Fora, MG, Brazil.; 4 Centro Interdisciplinar de Estudos e Pesquisas em Nefrologia, Juiz de Fora, MG, Brazil.; 5 IMUNOLAB Transplantes, Belo Horizonte, MG, Brazil.

**Keywords:** Renal Insufficiency, Chronic, Unified Health System, Health Care Coordination and Monitoring, Health Management, Decision Support Systems, Clinical

## Abstract

**Background:**

Appropriate referral and management of patients with chronic kidney disease (CKD) from primary care to nephrologists are essential to prevent disease progression and complications.

**Objectives:**

To describe the demographic and follow-up characteristics of patients with CKD stage 3b at the time of nephrology referral, the medical care offered by general practitioners, and the initial care provided by nephrologists in the Brazilian Unified Health System (SUS).

**Methods:**

This retrospective cohort study included all patients with CKD stage 3b on the waiting list for nephrology consultation between January 2018 and January 2020 in a large city in southern Brazil. Sociodemographic data, comorbidities, and adherence to follow-up recommendations based on the 2021 Kidney Disease: Improving Global Outcomes (KDIGO) guidelines were analyzed. Laboratory test requests and results were compared between the periods of general practitioner care and the period following nephrology consultation.

**Results:**

A total of 211 patients (mean age 74 ± 12 years) were included; 76.0% were hypertensive and 46.9% had diabetes. Approximately half had an adequate number of primary care appointments. Laboratory monitoring was suboptimal in both primary and specialized care. Before nephrology consultation, hemoglobin and urinalysis were available for 32.2% and 19.4% of patients, respectively. After consultation, hemoglobin and potassium results were documented in 68.3% and 58.8% of patients, respectively.

**Conclusion:**

Although patients with CKD stage 3b were regularly followed, low adherence to KDIGO testing recommendations raises concerns about the adequacy of care. Strengthening communication and guideline implementation between primary care and nephrology services is crucial to improve CKD management within the SUS.

## Introduction

Chronic kidney disease (CKD) has a global prevalence of 13.4%, and its incidence has been increasing^1^. Associated with significant morbidity and mortality, CKD has a substantial impact on the healthcare system, especially when patients reach advanced stages and require renal replacement therapy (RRT)^2,3,4,5,6,7^. Treatment before this stage focuses on controlling underlying diseases, slowing disease progression, and preparing for RRT^8,9^. Over the last few decades, increased attention has been placed on public health strategies to improve the care of patients with CKD. The principles of care have been compiled by Kidney Disease: Improving Global Outcomes (KDIGO), which serves as a global reference^9^.

Current KDIGO guidelines suggest that patients with stages 1–3 of CKD should be treated in primary care, while patients with CKD stages 4 and 5 should be referred for specialist care^9^. However, complications resulting from the loss of renal function can be present at stage 3b, necessitating therapeutic measures to manage these complications and slow disease progression even at this early stage^8,9,10,11,12^. General practitioners often experience doubts and anxieties regarding CKD management, leading to inadequate nephrology referrals and suboptimal patient care^13,14,15,16^. A well-organized and operational regulatory health system may address these issues^17^. The Brazilian Public Health System (SUS) provides universal healthcare access to the population, including care for patients with CKD prior to dialysis and all types of RRT (hemodialysis, peritoneal dialysis, and kidney transplantation). For patients with CKD stages 4 and 5, the system recommends referral to a nephrologist and multiprofessional care. For those with CKD stages 1–3, multidisciplinary matrix support is suggested, enabling the general practitioner to provide combined care with a nephrologist through teleconsultations conducted by telephone or e-mail^18,19^. However, implementing such a complex system is challenging in settings with large patient populations^19^.

Given the complexity of caring for patients with stage 3 CKD, it is important to evaluate whether general practitioners are aware of and adhere to the KDIGO recommendations for managing CKD^9^. This study aimed to evaluate whether general practitioners and nephrologists adhered to the KDIGO guidelines for managing patients with CKD stage 3b by assessing the number of appointments, laboratory test requests, and renin–angiotensin–aldosterone system blocker (RAASi) prescriptions.

## Methods

### Study Design

This was a retrospective cohort study of a convenience sample of patients with CKD stage 3b referred for nephrology care in a universal public healthcare system (SUS).

### Participants and Study Location

We studied patients with CKD stage 3b referred for nephrology care in the city of Belo Horizonte (Minas Gerais, Brazil) between January 2018 and January 2020. The city, as the state capital, provided specialized healthcare for a population of 2,521,564 inhabitants at the time of the study (IBGE)^20^. Data were collected from the regulatory system’s electronic records (referral data) and from electronic medical records (clinical and laboratory data) at the time of referral and for up to five years thereafter. The first available laboratory test in the electronic medical records was extracted both from the referral period and during nephrology care for up to five years. RAASi prescriptions were collected once, before nephrology care. The number of medical appointments per year before the nephrologist’s consultation was also recorded.

**Figure 1:**
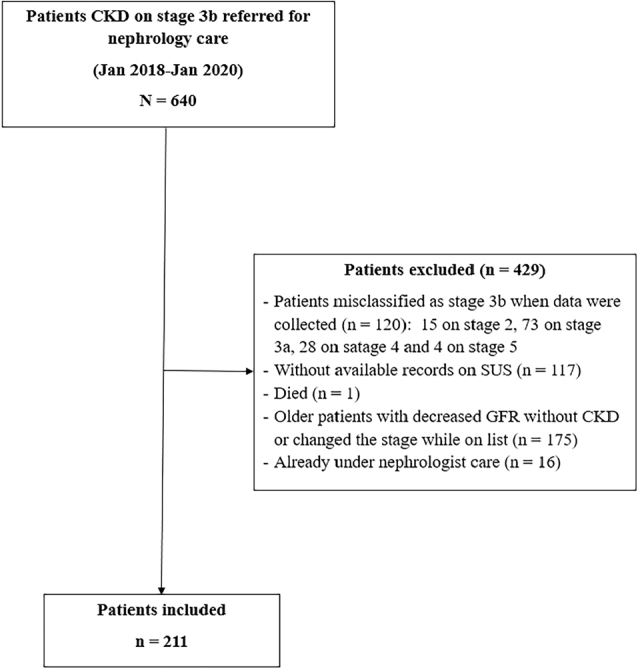
Study sample, inclusion, and exclusion criteria. SUS: Brazilian Public Health System.

Patients were eligible if they were at CKD stage 3b [estimated glomerular filtration rate (eGFR) of 30–44 mL/min/1.73 m^2^ using the CKD-EPI (Chronic Kidney Disease Epidemiology Collaboration) equation] when registered for nephrology consultation in the regulatory system and if they were seen by a nephrologist at least twice during the five years of the study. Exclusion criteria were: i) patients misclassified as stage 3b at the time of referral; ii) patients who were not followed in the accessible SUS electronic record system; iii) patients who were already under nephrology care but were mislabeled as first appointments; iv) those who died or started dialysis before their nephrology appointment; and v) patients excluded from the waiting list by the regulatory system because they changed stage while on the list or due to advanced age with decreased eGFR but without CKD complications (Figure 1).

Patients with CKD stage 3b were referred to the nephrology department in Belo Horizonte, MG, between January 2018 and January 2020. This study was approved by the Research Ethics Committees of the Faculty of Medical Sciences (CAAE 52619621.2.0000.5134) and the Municipal Health Secretariat of Belo Horizonte (CAAE 52619621.2.3001.5140) and was conducted in accordance with the Declaration of Helsinki. As the study was based on electronic records, the requirement for informed consent was waived.

### Variables and Data Collection

We assessed sociodemographic variables, including sex (male or female), age (years), level of education (years of schooling), self-declared ethnicity (yellow, white, mixed, and black), and comorbidities (diabetes mellitus and hypertension). We also collected laboratory data related to comorbidities, including LDL and HDL (mg/dL), uric acid (mg/dL), and sodium (mEq/L).

Renal function was assessed by eGFR, using the CKD-EPI equation, and by predictive variables for severity, such as the urine albumin-to-creatinine ratio (UACR, mg/g of creatinine). Laboratory tests (potassium levels in mEq/L), organic involvement secondary to loss of renal function [bone disease—parathyroid hormone (PTH) in pg/mL, calcium and phosphorus levels in mg/dL], and anemia [hemoglobin (Hb) in g/dL and the nutritional parameter albumin in g/dL] were also considered.

Adherence to KDIGO clinical guidelines^9,21^ was assessed by checking the frequency of medical consultations: two to three times annually if UACR was below 30 mg/g and three times annually if UACR was above 30 mg/g^22^. For laboratory tests, we evaluated the results available in laboratory or medical records. These results were considered adequate if found as follows: potassium, creatinine, UACR, and routine urinalysis, expected twice a year; Hb, PTH, and albumin, expected annually; and vitamin D, assessed at baseline^23,24^. This assessment was performed before and after nephrology consultation. Lipid, uric acid, and sodium profiles were also collected from medical and laboratory records, when available at least once. The prescription of RAASi was verified in the prescription section of the medical record at the time of referral.

### Regulation of Access to Nephrological Care

In Belo Horizonte, the capital of the third-largest state in Brazil, located in the Southeast region, more than thirteen thousand patients were referred by primary care to a nephrologist and placed on the waiting list in 2018. Access to a specialist occurs exclusively upon a primary care request, and all appointments are regulated and offered by a central regulation office. Following the regulatory protocols, patients with CKD stages 4 and 5, with massive proteinuria and a single kidney with nephrolithiasis, were seen by a nephrologist within 15 days of referral, representing approximately 10% of total referrals. The remaining patients were placed on a waiting list. Five nephrologists reviewed each case on this list, checking information from the referral request, medical records, and laboratory records, and found that 50% lacked the minimum data required to justify a nephrology appointment; these cases were placed on hold, and primary care was asked to complete the requisitions before the patient could be returned to the list. Twenty-two cases were outside the protocol and were removed from the system, with documentation informing the reason for this action. In total, 22% (2719 patients) remained on the waiting list for nephrology consultation, half of whom were in CKD stage 3b.

Although these patients were not considered to be under nephrologist care according to the guidelines, given that matrix support was not fully implemented in the city, the regulatory protocol determined that they should be under nephrological assistance—in this case, on the waiting list. Given the complexity of caring for patients with stage 3 CKD, it is important to evaluate whether general practitioners are aware of and follow KDIGO recommendations for managing CKD^9^. This is especially crucial due to the potential for significant and rapid declines in renal function and the high number of referrals requesting specialized assistance at this stage^22,25^.

### Statistical Analysis

Statistical analysis was performed using the Statistics and Data Science program Stata, version 17.0, with an α level of 0.05. Categorical variables were presented as absolute and relative frequencies. Continuous variables were described as means, standard deviations (SD), medians, and interquartile ranges, depending on the results of the Shapiro-Wilk test for normality. Differences between demographic variables were assessed using a binomial test for bimodal variables or a goodness-of-fit test for ordinal variables. The difference between pre- and post-nephrology examinations was determined by Fisher’s exact test for categorical variables or the Wilcoxon signed-rank test, considering the small sample size and non-normal distribution. The availability of laboratory test results before and after nephrology consultation was compared using risk ratios (RR), reported with 95% confidence intervals, considering the pre-nephrology group as the reference. Renal function was evaluated over the years using linear mixed models, with time as a numerical variable and participants as a random factor. Statistical significance was set at p < 0.05.

## Results

### Sociodemographic and Clinical Characteristics

A total of 211 eligible patients with a mean age of 74.0 ± 12 years were included, of whom 81.0% were older than 65 years (Figure 1). Most participants self-reported as non-Black, had a low level of education, and were overweight or obese (Table 1). Among the participants, 49.3% had diabetes mellitus, and 76.0% had hypertension. The average time to the nephrology consultation was 20 ± 4 months, ranging from 11 to 28 months.

**Table 1:** Sociodemographic and clinical characteristics of 211 patients with chronic kidney disease stage 3b

Variables	N = 211 patients	%	P value
*Sex*			
Male	114	54.0	0.2700^*^
Female	97	45.9	
*Ethnicity*			
Mixed	93	44.1	
White	84	39.8	
Black	31	14.7	
Yellow	3	1.4	
*Ethnicity*, 2 groups			
White	180	85.3	<0.0001^*^
Non-White	31	14.7	
*Years of schooling*
0–4 years	55	26	
4–8 years	125	59.3	
8–11 years	29	13.7	
>11 years	2	1	
*Education, 2 groups*			
Until elementary school	180	85.3	<0.0001^*^
Above elementary school	31	14.7	
*BMI*			
Eutrophic (18.5–24.9)	36	34.9	
Overweight (25–29.9)	31	30.1	
Obesity (>30)	36	34.9	
Missing data
*BMI*, 2 groups	108	51.2	
Eutrophic	36	35.0	0.0029^*^
Overweight and obesity	67	65.0	
*Diabetes Mellitus*	104	49.3	
*Hypertension*
*Prescription of RAASi*	161	76.0	
ARB	99	48.3	<0.0001^*^
ACEI	65	31.7	
None	47	22.8	

Abbreviations – BMI: Body Mass Index; RAASi: Renin–Angiotensin–Aldosterone System Blockers; ARB: Angiotensin Receptor Blocker; ACEI: Angiotensin–Converting Enzyme Inhibitor.

Note – *Binominal tests and goodness-of-fit test.

### Kdigo Adherence

Adherence to at least two medical consultations per year for this stage of CKD was achieved in 55.5% of patients receiving general practitioner care and in 49.3% of those followed up by a nephrologist. At the first consultation with the nephrologist, only 64.5% of patients underwent a creatinine testing, and only three had proteinuria results. Urinalysis was rarely available, being documented in only 19.4% (41) of patients before nephrological care and in 49.3% (104) after nephrology consultation (RR 1.59;1.37-1.84) (Table 2). Albuminuria data, assessed by UACR, were requested in only 1.4% (3) of initial consultations, increasing in subsequent years from 3.3% in the first year to 20.9% over five years of nephrology follow-up. Among diabetic patients, 71.1% (73) had glycated hemoglobin results.

**Table 2:** Availability of laboratory test results for 211 patients with chronic kidney disease stage 3b before and after nephrology consultation

Variable	Pre-Nephrologist	Post-Nephrologist	RR	IC	p-value
	N (%)	N (%)			
*Hemoglobin (g/dL)*	68 (32.2%)	144 (68.3%)	2.13	1.72–2.66	<0.001
*Albumin (g/dL)*	11 (5.2%)	72 (34.1%)	1.44	1.30–1.59	<0.001
*Potassium (mEq/L)*	24 (11.4%)	124 (58.8%)	2.15	1.82–2.54	<0.001
*Sodium (mEq/L)*	25 (11.9%)	71 (33.7%)	1.33	1.19–1.48	<0.001
*Calcium (mg/dL)*	16 (7.6%)	99 (46.9%)	1.74	1.53–1.99	<0.001
*Phosphorus (mg/dL)*	12 (5.7%)	82 (38.9%)	1.54	1.38 –1.73	<0.001
*Vitamin D (ng/mL)*	25 (11.8%)	104 (49.3%)	1.74	1.51–2.00	<0.001
*PTH (pg/mL)*	6 (2.8%)	31 (14.7%)	1.14	1.07–1.21	<0.001
*Uric acid (mg/dL)*	37 (17.5%)	106 (50.2%)	1.66	1.43–1.92	<0.001
*LDL (mg/dL)*	20 (9.5%)	77 (36.5%)	1.43	1.28–1.59	<0.001
*HDL (mg/dL)*	11 (5.2%)	53 (25.1%)	1.27	1.16–1.38	<0.001
*Triglycerides (mg/dL)*	17 (8.1%)	69 (21.7%)	1.37	1.23–1.51	<0.001
*Routine urine*	41 (19.4%)	104 (49.3%)	1.59	1.37–1.84	<0.001

Renal function, measured using serum creatinine levels, was reported in 136 (64.5%) patients at the time of request. Regarding records per year, this parameter was observed in 31 (14.7%) patients in 2018, 118 (55.9%) in 2019, and 82 (38.9%) in 2020. These data were collected overall, including subjects who had already undergone a nephrology consultation and others who were still awaiting consultation between 2018 and 2020. Renal function assessments were also performed in 124 (58.8%) and 45 (21.3%) individuals under nephrologist care in 2021 and 2022, respectively (Table 3).

**Table 3:** Evolution of glomerular filtration rate, calculated from creatinine using the ckd-epi equation, from referral to the nephrologist until 2022

Year	Referrals – N (%)	Mean GFR (mL/min/1.73 m^2^)	P value	Coefficient
Referral	136 (64.45%)	38.64 ± 8.41		
2018	31 (14.69%)	38.95 ± 7.7	0.733	0.57
2019	118 (55.92%)	39.77 ± 11.23	0.217	1.25
2020	82 (38.86%)	37.42 ± 8.8	0.439	–0.88
2021	124 (58.77%)	35.04 ± 8.96	0.001	–3.35
2022	45 (21.33%)	23.48 ± 7.31	0.000	–15.49

Abbreviation – GFR: Glomerular filtration rate.

**Figure 2:**
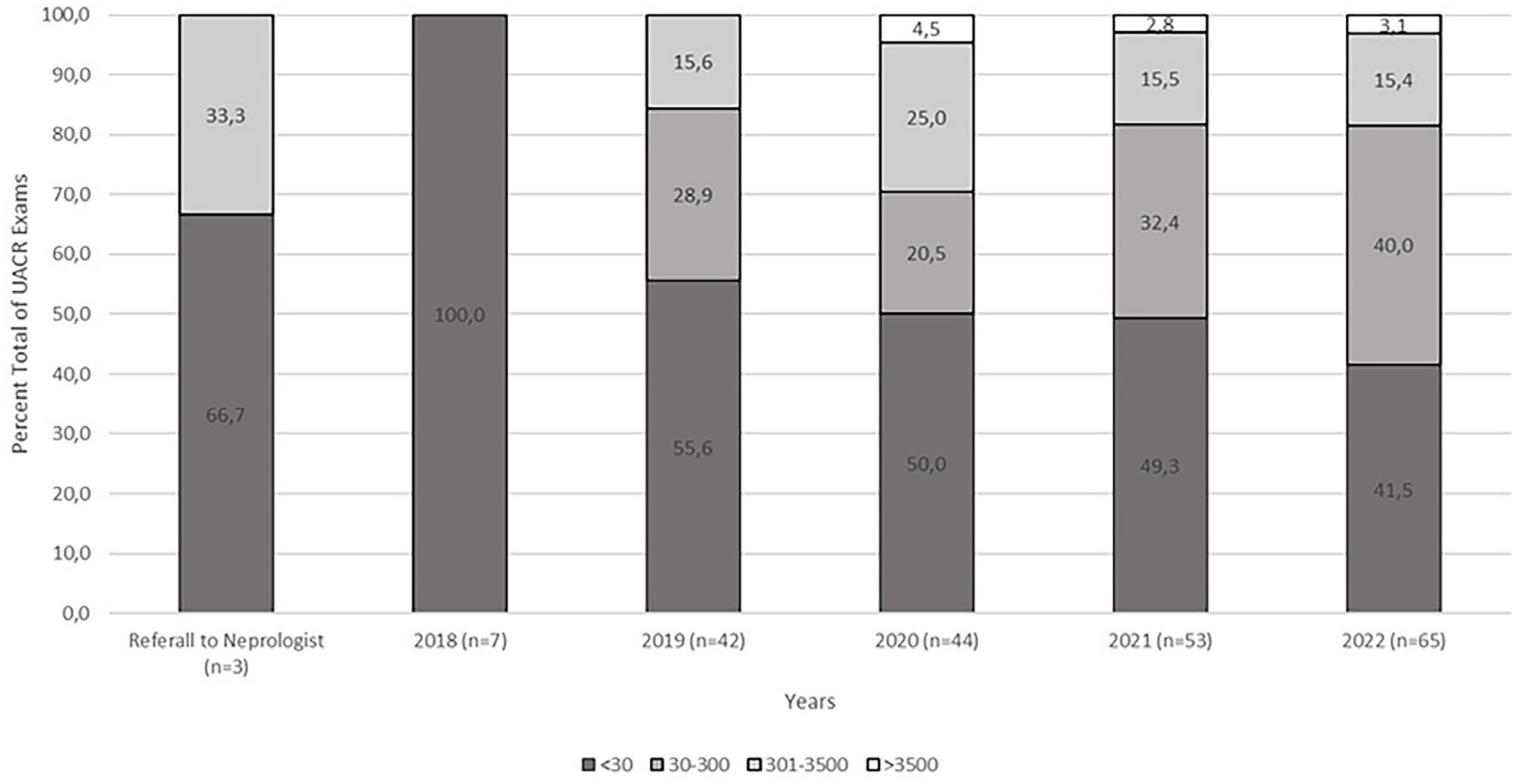
Albuminuria levels measured by the UACR (mg/g of creatinine) at referral and after nephrology care.

Albuminuria assessments, such as UACR or routine urine tests, were rarely requested, even among the 76% of patients with hypertension and the 49.3% with diabetes mellitus. Although UACR > 300 mg/g was observed in some patients, the highest incidence occurred in 2020, with only 11 (25%) results available (Figure 2).

Eighty percent of the participants were receiving RAASi therapy, 48.3% were using angiotensin receptor blockers, and 31.7% were taking angiotensin-converting enzyme inhibitors (Table 1).

The proportion of patients with available laboratory results for calcium, phosphorus, albumin, vitamin D, parathyroid hormone (PTH), uric acid, lipid profile, and sodium, tests recommended by clinical guidelines for patients with stage 3b disease, ranged from 5% to 32% prior to the nephrology consultation (Table 2). After nephrology care, these tests were requested and available for 25–50% of patients, except for hemoglobin and potassium, which were available for 68% and 58.8%, respectively (Table 2).

### Complications Resulting from Loss of Renal Function

The mean levels and the percentage of tests outside the optimal values defined by the guidelines did not differ between pre- and post-nephrology care (Table 4). Serum potassium levels were higher than 5 mEq/L in 30% of patients in both groups (highest value of 6.5 mEq/L), even though few examinations were requested. Anemia, defined as Hb < 12 g/dL, was observed in 43.6% and 36.1% of patients pre- and post-nephrology care, respectively (Table 4). Anemia with an indication for therapeutic intervention (Hb < 10 g/dL) was less frequent (7.4% and 4.9% in patients pre- and post-nephrology care, respectively).

**Table 4:** Laboratory test results for the 211 patients with chronic kidney disease stage 3b before and after nephrology consultation

Variables	Pre-Nephrologist N (%)	Post-Nephrologist N (%)	P value
*Hemoglobin (g/dL)*
Min–Max	9.1–17.9	8.4–17.4	
Mean (SD)	12.45 ± 1.80	12.82 ± 1.79	
Stratification
Hemoglobin <10	5 (7.4%)	7 (4.9%)	
Hemoglobin 10–12	26 (38.2%)	45 (31.3%)	0.001^*^
Hemoglobin >12	37 (54.4%)	92 (63.8%)	
Total	68 (100%)	144 (100%)	
Cardiovascular disease	17 (8%)	32 (15.2%)	
Association with risk factors	0	0	
Hemoglobin <10 cardiovascular			0.5200^*^
Cancer	9 (4.3%)	14 (6.6%)
Hemoglobin <10 cancer	1 (0.5%)	1 (0.5%)	
*Albumin (g/dL)*
Min–Max	2.9–4.9	2.6–4.8	
Mean (SD)	4.02 ± 0.53	4.12 ± 0.42	
Stratification
Albumin <3.5	1 (9.1%)	4 (5.6%)	0.1430^*^
Albumin >3.5	10 (90.9%)	68 (94.4%)	
Total	11 (100%)	72 (100%)	
*Potassium (mEq/L)*
Min–Max	3.5–6.2	3.7–6.5	
Mean (SD)	4.76 ± 0.72	4.80 ± 0.51	
Stratification
Potassium <3.5	0	0	
Potassium 3.5–5	17 (70.8%)	87 (70.7%)	0.2080^*^
Potassium >5	7 (29.2%)	37 (29.8%)	
Total	24 (100%)	124 (100%)	
*Sodium (mEq/L)*
Min–Max	136–148	126–145	
Mean (SD)	139.96 ± 3.59	139.69 ± 2.95	0.4413^*^
Stratification
Sodium <135	0	4 (5.6%)	
Sodium 135–145	22 (88.0%)	67 (94.4%)	NA
Sodium >145	3 (12.0%)	0	
Total	25	71 (100%)	
*Calcium (mg/dL)*			
Min–Max	8.2–10.7	4.7–11.6	
Mean (SD)	9.37 ± 0.59	9.36 ± 0.75	0.7649^*^
Stratification			
Calcium <8.5	1 (6.3%)	5 (5.1%)	
Calcium 8.5–10.5	14 (87.5%)	92 (92.9%)	NA
Calcium >10.5	1 (6.3%)	2 (2.0%)	
Total	16 (100%)	99 (100%)	
*Phosphorus (mg/dL)*			
Min–Max	2.9–4.5	2.4–6.6	
Mean (SD)	3.66 ± 0.47	3.58 ± 0.71	0.9164^*^
Stratification			
Phosphorus <2.5	0	1 (1.2%)	
Phosphorus 2.5–5.6	12 (100%)	80 (97.6%)	NA
Phosphorus >5.6	0	1 (1.2%)	
Total	12 (100%)	82 (100%)	
*Vitamin D (ng/mL)*			
Min–Max	15–58	8–61	
Mean (SD)	30.76 ± 11.31	27.00 ± 11.27	0.6097^*^
Stratification			
Vitamin D <30	12 (48.0%)	59 (56.7%)	1^*^
Vitamin D >30	13 (52.0%)	45 (43.3%)	
Total	25 (100%)	104 (100%)	
*PTH (pg/mL)*			
Min–Max	14–286	12.4–224	
Mean (SD)	98.5 ± 96.98	88.92 ± 64.73	
Stratification 1			
PTH <80	4 (66.7%)	20 (64.5%)	NA
PTH >80	2 (33.3%)	11 (35.5%)	
Total	6 (100%)	31 (100%)	
Stratification 2			
PTH <150	5 (83.3%)	25 (80.7%)	NA
PTH >150	1 (16.7%)	6 (19.4%)	
Total	6 (100%)	31 (100%)	
*Uric acid (mg/dL)*			
Min–Max	2–11.8	3.3–12	
Mean (SD)	7.3 ± 2.1	7.01 ± 1.62	0.6681^*^
Stratification			
Uric acid <6	8 (21.6%)	28 (26.4%)	0.6010^*^
Uric acid 6–7	4 (10.8%)	20 (18.9%)	
Uric acid >7	25 (67.6%)	58 (54.7%)	
Total	37 (100%)	106 (100%)	
*LDL (mg/dL)*			
Min–Max	55–170	44–273	
Mean (SD)	92.45 ± 27.01	107.37 ± 43.72	0.6733^*^
Stratification			
LDL <100	14 (70.0%)	38 (49.4%)	0.4670^*^
LDL >100	6 (30.0%)	39 (50.7%)	
Total	20(100%)	77 (100%)	
*HDL (mg/dL)*			
Min–Max	29–73	26–97	
Mean (SD)	43.91 ± 12.87	50.23 ± 17.45	0.8527^*^
Stratification			
HDL <35	4 (36.4%)	10 (8.9%)	NA
HDL >35	7 (63.6%)	43 (81.1%)	
Total	11 (100%)	53 (100%)	
*Triglycerides (mg/dL)*			
Min–Max	37–1357	39–2021	
Mean (SD)	353.76 ± 391.09	187.01 ± 242.41	0.7532^*^
Stratification			
Triglycerides <150	5 (29.4%)	36 (52.2%)	NA
Triglycerides >150	12 (70.6%)	33 (47.8%)	
Total	17 (100%)	69 (100%)	
*Routine urine*			
Normal	32 (54.4%)	71 (68.3%)	
Glucosuria	1 (2.4%)	3 (2.9%)	
Pyuria	1 (2.4%)	1 (1.0%)	
Hematuria	3 (7.3%)	3 (2.9%)	
Albuminuria (+)	2 (4.9%)	19 (18.3%)	
Albuminuria (+ +)	1 (2.4%)	6 (5.8%)	
Albuminuria (+ + +)	1 (2.4%)	1 (1.0%)	
Total	41 (100%)	104	

Abbreviations – Min–Max: minimum–maximum; NA: Not applicable; PTH: Parathyroid hormone; SD: Standard deviation.

Notes – *Wilcoxon signed-rank and Fisher’s exact tests. Reference ranges from the local laboratory for phosphorus, as suggested by KDIGO.

### Renal Function Decline

The linear mixed model demonstrated that no significant decline in renal function was observed, as measured by eGFR, during the pre-nephrology care period. However, renal function declined after the initiation of nephrology care, with reductions at 4 (3.25 mL/min) and at 5 years (15.49 mL/min) after referral (Table 3).

## Discussion

Our results indicate that general practitioners infrequently adhere to KDIGO guidelines for the referral of patients to a nephrologist or for the adequate follow-up of recommended laboratory tests. Nevertheless, these professionals play an important role in the comprehensive management of chronic kidney disease. Their contribution extends beyond the early detection and monitoring of renal impairment, encompassing the prevention of hospitalizations and the coordination of multidisciplinary care. Previous studies have demonstrated that general practitioner involvement can reduce the risk of hospitalization, even during challenging periods such as the COVID-19 pandemic^26,27,28^.

The average time to the first nephrology appointment was 20 months, and most of the patients were seen 2–3 times per year, in line with the guidelines, either before or after nephrology care. Notably, there were no differences between pre- and post-nephrology care in the frequency of CKD complications, such as anemia, hyperkalemia, and hypocalcemia. However, a decline in eGFR was observed when comparing the last two years with baseline data.

In patients with CKD, the anticipated GFR loss is reported to be between 0.3 and 1 mL/min/year^8,24,29,30^, which aligns with our findings in the first three years of follow-up. Nevertheless, we observed an accelerated decline in renal function in the last four and five years, exceeding the 5 mL/min/year threshold suggested by KDIGO. It remains unclear whether the observed decline in renal function was related to a reduced frequency of routine medical appointments, limited access to laboratory testing, or the direct or indirect effects of SARS-CoV-2 infection. However, given the retrospective design of this study, it was not possible to control for potential confounding factors or to establish a causal relationship between the pandemic and the observed decline in renal function. This limitation should be acknowledged and considered when interpreting these findings^1,31^.

The Chronic Kidney Disease Outcomes and Practice Patterns Study (CKDopps)^32^ evaluated pre-dialysis patients under nephrology care in various countries, including 888 patients from Brazil; however, only 31 were in stage 3b. Our study, in contrast, focused on patients initially managed in primary care, highlighting current gaps in nonspecialized care for this population. Notably, the CKDopps cohort was 10 years younger than our study population, even though we included only patients with CKD stage 3b.

We observed a concerning lack of availability of laboratory test results for patients with CKD under general practitioner or nephrologist care. Less than 25% of tests recommended by the guidelines were available in our study, similar to that reported in the CKDopps^32^, where albuminuria results were available for less than half of the study population. It remains unclear whether this is due to physicians’ unawareness of the guidelines or a failure by participants to complete the requested tests.

Comorbidities such as diabetes and hypertension are prevalent in this patient population and require adequate control and follow-up^33,34^. Even UACR, a routine examination, was rarely available for our study participants. The prevalence of hypertension in patients with CKD stage 3b was consistent with previous reports^35,36^. Anemia, defined as Hb < 12 g/dL, was also prevalent despite nephrology care. However, in Brazil, the treatment of anemia in patients with CKD using erythropoietin and iron supplementation is provided by the SUS only when the Hb level is lower than 10 g/dL^36^, which corresponded to less than 10% of patients before and after nephrology care.

This observational study is the first to track patients with CKD stage 3b managed in primary care. We included all eligible patients on the waitlist at the time of the study. Additionally, the integrated SUS system provides a unique opportunity to understand multiple aspects of care for these patients within the public healthcare network (e.g., access to consultations and laboratory test results). However, as a retrospective study, the quality and completeness of data extracted from electronic medical records may have affected the consistency of the analyses and the robustness of the conclusions. The limited availability of laboratory data highlights a major challenge in ensuring adequate patient follow-up. It remains uncertain whether these tests were not requested by healthcare providers or were not completed by patients, which warrants further investigation. Furthermore, because the study relied on a convenience sample restricted to a single city, the findings may not be generalizable to other Brazilian regions that differ in healthcare structure, resource distribution, and population characteristics. Despite these limitations, the study accurately portrays real-world CKD management in primary care and emphasizes persistent barriers related to adherence to clinical guidelines and coordination across healthcare levels. The results help identify gaps that may guide strategies to enhance clinical practice and healthcare management for CKD patients.

## Conclusion

Although patients with CKD stage 3b were seen frequently by their physicians, the lack of laboratory tests, as recommended by the KDIGO, raises concerns about the adequacy of care provided. Conversely, no significant decline in renal function was observed in the first two years, which corresponded to the median time to nephrology consultation. However, insufficient monitoring of key complications, such as albuminuria, anemia, and mineral and bone disorders, indicates that the absence of a decline in eGFR alone cannot be interpreted as evidence of clinical safety during this waiting period. Future research should investigate the impact of delayed nephrology consultation on the progression of CKD and its complications within the primary care system, particularly in the context of nephrological matrix support. Additionally, efforts should focus on improving the implementation of clinical guidelines and understanding the barriers to their adherence. These findings highlight the need for continuous professional education and structured matrix support within the SUS to enhance the quality of care for patients with CKD stage 3b.

## Data Availability

Data will be made available on request.
