# Supplementary material for: Patients with chronic kidney disease stage 3b: do general practitioners and nephrologists follow KDIGO guidelines?
Source: J Bras Nefrol. 2026 Apr 17;48(3):e20250229. doi: 10.1590/2175-8239-JBN-2025-0229en (PMC13101827; doi:10.1590/2175-8239-JBN-2025-0229en)
Supplement: Translation

# Translation: Pacientes com doença renal crônica estágio 3b: médicos generalistas e nefrologistas seguem as diretrizes da KDIGO?

**DOI:** 10.1590/2175-8239-JBN-2025-0229pt

**Authors:** Juliana Gazzi Macedo, Alessandra Maciel Almeida, Helady Sanders-Pinheiro, Raquel Aparecida Fabreti-Oliveira

**Affiliations:** 1 Faculdade de Ciências Médicas de Minas Gerais, Belo Horizonte, MG, Brasil.; 2 Universidade Federal de Minas Gerais, Belo Horizonte, MG, Brasil.; 3 Universidade Federal de Juiz de Fora, Hospital Universitário, Unidade de Transplante Renal, Juiz de Fora, MG, Brasil.; 4 Centro Interdisciplinar de Estudos e Pesquisas em Nefrologia, Juiz de Fora, MG, Brasil.; 5 IMUNOLAB Transplantes, Belo Horizonte, MG, Brazil.

**Keywords:** Insuficiência Renal Crônica;, Sistema Único de Saúde;, Regulação e Fiscalização em Saúde;, Gestão em Saúde;, Sistemas de Apoio a Decisões Clínicas

## Abstract

**Introdução:**

O encaminhamento e o manejo adequados de pacientes com DRC da atenção primária à nefrologia são fundamentais para prevenir complicações e a progressão da doença.

**Objetivos:**

Descrever características demográficas e de acompanhamento de pacientes com DRC estágio 3b no momento do encaminhamento, o cuidado oferecido por generalistas e a assistência inicial por nefrologistas no Sistema Único de Saúde (SUS).

**Métodos:**

Coorte retrospectiva incluindo todos os pacientes com DRC estágio 3b na lista de espera para consulta nefrológica entre janeiro de 2018 e janeiro de 2020, em uma grande cidade do sudeste do Brasil. Foram avaliados dados sociodemográficos, comorbidades e adesão às recomendações de acompanhamento segundo *Kidney Disease: Improving Global Outcomes* (KDIGO) 2021. Os pedidos e resultados de exames laboratoriais foram comparados entre os períodos de atendimento pelo clínico geral e após consulta nefrológica.

**Resultados:**

Um total de 211 pacientes (idade média de 74 ± 12 anos) foram incluídos; 76% eram hipertensos e 46,9%, diabéticos. Cerca de metade realizou número adequado de consultas na atenção básica. O monitoramento laboratorial foi subótimo tanto na atenção primária quanto no cuidado especializado. Antes da nefrologia, exames de dosagem de hemoglobina e exames de urina estavam disponíveis em 32,2% e 19,4% dos pacientes, respectivamente. Após a consulta especializada, hemoglobina e potássio foram registrados para 68,3% e 58,8% dos pacientes, respectivamente.

**Conclusão:**

Embora os pacientes com DRC estágio 3b fossem acompanhados regularmente, a baixa adesão às recomendações de testes da KDIGO levanta preocupações quanto à adequação do cuidado. Fortalecer a comunicação e a implementação das diretrizes entre a atenção primária e os serviços de nefrologia é fundamental para aprimorar o manejo da DRC no âmbito do SUS.

## Introdução

A doença renal crônica (DRC) tem uma prevalência global de 13,4%, e sua incidência vem aumentando^1^. Associada à significativa morbimortalidade, a DRC exerce substancial impacto sobre o sistema de saúde, especialmente quando os pacientes atingem estágios avançados e necessitam de terapia renal substitutiva (TRS)^2,3,4,5,6,7^. O tratamento antes dessa fase concentra-se no controle das doenças de base, na desaceleração da progressão da doença e na preparação para a TRS^8,9^. Nas últimas décadas, estratégias de saúde pública têm recebido maior atenção para aprimorar o cuidado prestado a pacientes com DRC. Os princípios de cuidado foram compilados pela *Kidney Disease: Improving Global Outcomes* (KDIGO), que serve como referência internacional^9^.

As diretrizes atuais da KDIGO sugerem que pacientes com DRC nos estágios 1–3 devem ser tratados na atenção primária, enquanto pacientes com DRC nos estágios 4 e 5 devem ser encaminhados ao atendimento especializado^9^. Contudo, complicações decorrentes da perda de função renal podem estar presentes no estágio 3b, exigindo medidas terapêuticas para manejar essas complicações e retardar a progressão da doença ainda nessa fase inicial^8,9,10,11,12^. Médicos generalistas frequentemente apresentam dúvidas e inseguranças quanto ao manejo da DRC, resultando em encaminhamentos inadequados à nefrologia e em cuidados subótimos aos pacientes^13,14,15,16^. Um sistema de regulação em saúde bem organizado e funcional pode mitigar esses problemas^17^. O Sistema Único de Saúde (SUS) brasileiro oferece acesso universal à saúde para a população, incluindo cuidados para pacientes com DRC antes da diálise e todos os tipos de TRS (hemodiálise, diálise peritoneal e transplante renal). Para pacientes nos estágios 4 e 5, o sistema recomenda encaminhamento ao nefrologista e acompanhamento multiprofissional. Para aqueles nos estágios 1–3, sugere-se apoio matricial multidisciplinar, permitindo que o médico generalista preste atendimento conjunto com o nefrologista por meio de teleconsultorias por telefone ou e-mail^18,19^. Entretanto, implementar um sistema tão complexo é um desafio em locais com grande volume de pacientes^19^.

Dada a complexidade do cuidado de pacientes com DRC estágio 3, é essencial avaliar se os médicos generalistas conhecem e seguem as recomendações da KDIGO para o manejo da doença^9^. Este estudo teve como objetivo avaliar se médicos generalistas e nefrologistas aderiram às diretrizes da KDIGO no manejo de pacientes com DRC estágio 3b, analisando o número de consultas, solicitações laboratoriais e prescrições de inibidores do sistema renina-angiotensina-aldosterona (iSRAA).

## Métodos

### Desenho do Estudo

Trata-se de um estudo de coorte retrospectivo com uma amostra de conveniência de pacientes com DRC estágio 3b encaminhados para atendimento nefrológico em um sistema público de saúde de acesso universal–o SUS.

### Participantes e Local do Estudo

Foram incluídos pacientes com DRC estágio 3b encaminhados para atenção nefrológica na cidade de Belo Horizonte (Minas Gerais, Brasil) entre janeiro de 2018 e janeiro de 2020. A cidade, capital do estado, oferecia atendimento especializado para uma população de 2.521.564 habitantes na ocasião do estudo, segundo o IBGE^20^. Os dados foram coletados a partir dos registros eletrônicos do sistema de regulação (dados de encaminhamento) e de prontuários eletrônicos (dados clínicos e laboratoriais) no momento do encaminhamento e até cinco anos após.

O primeiro exame laboratorial disponível nos prontuários eletrônicos foi extraído tanto do período de encaminhamento quanto durante o acompanhamento nefrológico por até cinco anos. As prescrições de iSRAA foram coletadas uma única vez, antes do atendimento nefrológico. O número de consultas médicas anuais antes da consulta com o nefrologista também foi registrado.

Foram elegíveis pacientes classificados como estágio 3b [taxa de filtração glomerular estimada (TFGe) de 30–44 mL/min/1,73 m^2^ pela equação CKD-EPI (*Chronic Kidney Disease Epidemiology Collaboration*)], registrados no sistema de regulação para consulta nefrológica e atendidos por um nefrologista pelo menos duas vezes durante os cinco anos do estudo. Os critérios de exclusão foram: i) pacientes erroneamente classificados como estágio 3b no pedido; ii) pacientes não acompanhados pelo sistema eletrônico acessível do SUS; iii) pacientes já em acompanhamento nefrológico, mas registrados como solicitação de primeira consulta; iv) pacientes que faleceram ou iniciaram diálise antes da consulta nefrológica; v) pacientes excluídos da lista de espera pela regulação devido à mudança de estágio ou por apresentarem idade avançada, com TFGe reduzida, mas sem complicações da DRC (Figura 1).

**Figura 1:** Amostra do estudo, critérios de inclusão e exclusão. SUS: Sistema Único de Saúde do Brasil. DRC: Doença renal crônica; TFG: Taxa de filtração glomerular.

Pacientes com DRC estágio 3b foram encaminhados ao serviço de nefrologia em Belo Horizonte, MG, entre janeiro de 2018 e janeiro de 2020. Este estudo foi aprovado pelo Comitê de Ética em Pesquisa da Faculdade de Ciências Médicas (CAAE 52619621.2.0000.5134) e pela Secretaria Municipal de Saúde de Belo Horizonte (CAAE 52619621.2.3001.5140), sendo conduzido conforme a Declaração de Helsinque. Como o estudo utilizou registros eletrônicos, o consentimento informado foi dispensado.

### Variáveis e Coleta de Dados

Foram avaliadas variáveis sociodemográficas, incluindo sexo (masculino ou feminino), idade (anos), escolaridade (anos de estudo), etnia autodeclarada (amarela, branca, parda e preta) e comorbidades (diabetes mellitus e hipertensão arterial). Foram coletados dados laboratoriais relacionados às comorbidades, incluindo LDL e HDL (mg/dL), ácido úrico (mg/dL) e sódio (mEq/L).

A função renal foi avaliada pela TFGe, utilizando a equação CKD-EPI, e por variáveis preditoras de gravidade, como a razão albumina-creatinina urinária (RAC, mg/g de creatinina). Também foram considerados exames laboratoriais coletados (níveis de potássio em mEq/L), o comprometimento orgânico secundário à perda da função renal [doença óssea—paratormônio (PTH) em pg/mL, cálcio e fósforo em mg/dL] e anemia [hemoglobina (Hb) em g/dL e o parâmetro nutricional albumina em g/dL].

A adesão às diretrizes da KDIGO^9,21^ foi avaliada por meio da frequência de consultas médicas: duas a três vezes ao ano se RAC fosse menor que 30 mg/g, e três vezes ao ano se RAC fosse superior a 30 mg/g^22^. Para os exames laboratoriais, avaliamos os resultados disponíveis nos registros laboratoriais ou médicos. Esses resultados foram considerados adequados quando encontrados da seguinte forma: potássio, creatinina, RAC e urina de rotina, esperados duas vezes ao ano; Hb, PTH e albumina, esperados anualmente; e vitamina D, avaliada no momento basal^23,24^. Essa verificação foi realizada antes e após a consulta com o nefrologista. Os perfis lipídico, de ácido úrico e de sódio também foram coletados dos registros médicos e laboratoriais, quando disponíveis ao menos uma vez. A prescrição de iSRAA foi verificada na área de prescrição do prontuário no momento do encaminhamento.

### Regulação do Acesso ao Cuidado Nefrológico

Em Belo Horizonte, capital do terceiro maior estado do Brasil, situada na região Sudeste, mais de treze mil pacientes foram encaminhados pela atenção primária para a lista de espera de consulta com nefrologista no ano de 2018. O acesso ao especialista ocorre exclusivamente mediante solicitação da atenção primária, e todas as consultas são reguladas e distribuídas por uma central de regulação. De acordo com os protocolos de regulação, pacientes com DRC estágios 4 e 5, com proteinúria maciça e rim único com nefrolitíase, foram atendidos pelo nefrologista em até 15 dias após o encaminhamento, representando aproximadamente 10% de todos os encaminhamentos. Os demais pacientes foram colocados em lista de espera. Cinco nefrologistas revisaram cada caso dessa lista, verificando as informações da solicitação, dos prontuários médicos e de registros laboratoriais, e constataram que 50% não apresentavam dados mínimos para justificar a necessidade de consulta nefrológica; esses pacientes permaneceram em espera, e a atenção primária foi orientada a completar as requisições para recolocar o paciente na relação de encaminhamentos. Vinte e dois casos estavam fora do protocolo e foram removidos do sistema, com registro do motivo dessa ação. No total, 22% (2.719 pacientes) permaneceram na lista de espera para consulta com nefrologista, sendo que metade deles estava no estágio 3b da DRC.

Embora esses pacientes não fossem considerados sob cuidado nefrológico segundo as diretrizes, devido à implementação incompleta do apoio matricial na cidade, o protocolo regulatório entendeu que eles deveriam estar sob assistência nefrológica—neste caso, na lista de espera. Dada a complexidade do manejo de pacientes com DRC estágio 3, torna-se relevante avaliar se os médicos generalistas conhecem e seguem as recomendações da KDIGO para o tratamento da DRC^9^. Isso é particularmente crucial devido ao potencial declínio significativo e rápido da função renal e ao elevado número de encaminhamentos solicitando assistência especializada nesse estágio^22,25^.

### Análise Estatística

A análise estatística foi realizada utilizando o programa de Estatística e Ciência de Dados Stata, versão 17.0, com nível de significância α de 0,05. As variáveis categóricas foram apresentadas como frequências absolutas e relativas. As variáveis contínuas foram descritas por meio de médias, desvios-padrão (DP), medianas e intervalos interquartis, conforme o resultado do teste de normalidade de *Shapiro–Wilk*. Já as diferenças entre variáveis demográficas foram avaliadas por meio do teste binomial, para variáveis bimodais, ou do teste de aderência (*goodness of fit*), para variáveis ordinais. A diferença entre os exames realizados antes e após a consulta com a nefrologia foi determinada pelo teste exato de *Fisher*, para variáveis categóricas, ou pelo teste de *Wilcoxon* (*signed-ranks*), considerando o tamanho amostral reduzido e a distribuição não normal. A disponibilidade dos resultados de exames laboratoriais antes e após a consulta nefrológica foi comparada por meio de razões de risco (RR), apresentadas com seus respectivos intervalos de confiança de 95%, considerando o grupo pré-consulta nefrológica como referência. A função renal foi avaliada ao longo dos anos utilizando modelos lineares mistos, com o tempo como variável numérica e os participantes como fator aleatório. O nível de significância estatística foi estabelecido em p < 0,05.

## Resultados

### Características Sociodemográficas e Clínicas

Foram incluídos 211 pacientes elegíveis, com idade média de 74,0 ± 12 anos, dos quais 81,0% tinham mais de 65 anos (Figura 1). A maioria dos participantes se autodeclarou não negra, tinha baixo nível de escolaridade e apresentava sobrepeso ou obesidade (Tabela 1). Entre os participantes, 49,3% tinham diabetes mellitus e 76,0% tinham hipertensão arterial. O tempo médio até a consulta com o nefrologista foi de 20 ± 4 meses, variando entre 11 e 28 meses.

**Tabela 1:** Características sociodemográficas e clínicas de 211 pacientes com doença renal crônica estágio 3b

Variáveis	N = 211 pacientes	%	Valor de p
*Sexo*			
Masculino	114	54,0	0,2700^*^
Feminino	97	45,9	
*Etnia*			
Parda	93	44,1	
Branca	84	39,8	
Negra	31	14,7	
Amarela	3	1,4	
*Etnia*, 2 grupos
Branca	180	85,3	<0,0001^*^
Não-Branca	31	14,7	
*Anos de escolaridade*
0–4 anos	55	26	
4–8 anos	125	59,3	
8–11 anos	29	13,7	
>11 anos	2	1	
*Escolaridade, 2 grupos*
Até o Ensino Fundamental	180	85,3	<0,0001^*^
Acima do Ensino Fundamental
*IMC*	31	14,7	
Eutrófico (18,5–24,9)	36	34,9	
Sobrepeso (25–29,9)	31	30,1	
Obesidade (>30)	36	34,9	
Dados faltantes	108	51,2	
*IMC*, 2 grupos
Eutrófico	36	35,0	0,0029^*^
Sobrepeso e obesidade	67	65,0	
*Diabetes Mellitus*	104	49,3	
*Hipertensão*	161	76,0	
*Prescrição de bloqueadores do SRAA*			
BRA	99	48,3	<0,0001^*^
IECA	65	31,7	
Nenhum	47	22,8	

Abreviaturas – IMC: Índice de Massa Corporal; SRAA: Sistema Renina–Angiotensina–Aldosterona; BRA: Bloqueador do Receptor de Angiotensina; IECA: Inibidor da Enzima Conversora de Angiotensina.

Note – *Testes binomiais e teste de bondade de ajuste.

### Adesão às Diretrizes Kdigo

A adesão a pelo menos duas consultas médicas por ano para este estágio de DRC foi observada em 55,5% dos pacientes acompanhados na atenção primária e em 49,3% daqueles acompanhados pelo nefrologista. Na primeira consulta com o nefrologista, apenas 64,5% dos pacientes realizaram exame de creatinina, e somente três apresentaram resultados de proteinúria. O exame de urina simples esteve raramente disponível, presente em somente 19,4% (41) dos pacientes antes do atendimento nefrológico e em 49,3% (104) após o atendimento (RR 1,59; 1,37-1,84) (Tabela 2). Dados de albuminúria, avaliados por RAC, foram solicitados em apenas 1,4% (3) das consultas iniciais, aumentando nos anos seguintes, de 3,3% no primeiro ano para 20,9% ao longo de cinco anos de acompanhamento nefrológico. Entre os pacientes diabéticos, 71,1% (73) apresentaram resultados de hemoglobina glicada.

**Tabela 2:** Disponibilidade de exames laboratoriais em 211 pacientes com drc estógio 3b antes e após consulta nefrológica

Variável	Pré-Nefrologista	Pós-Nefrologista	RR	IC	Valor de p
	N (%)	N (%)			
Hemoglobina (g/dL)	68 (32,2%)	144 (68,3%)	2,13	1,72–2,66	<0,001
Albumina (g/dL)	11 (5,2%)	72 (34,1%)	1,44	1,30–1,59	<0,001
Potássio (mEq/L)	24 (11,4%)	124 (58,8%)	2,15	1,82–2,54	<0,001
Sódio(mEq/L)	25 (11,9%)	71 (33,7%)	1,33	1,19–1,48	<0,001
Cálcio (mg/dL)	16 (7,6%)	99 (46,9%)	1,74	1,53–1,99	<0,001
Fósforo (mg/dL)	12 (5,7%)	82 (38,9%)	1,54	1,38–1,73	<0,001
Vitamina D (ng/mL)	25 (11,8%)	104 (49,3%)	1,74	1,51–2,00	<0,001
PTH (pg/mL)	6 (2,8%)	31 (14,7%)	1,14	1,07–1,21	<0,001
Ácido úrico (mg/dL)	37 (17,5%)	106 (50,2%)	1,66	1,43–1,92	<0,001
LDL (mg/dL)	20 (9,5%)	77 (36,5%)	1,43	1,28–1,59	<0,001
HDL (mg/dL)	11 (5,2%)	53 (25,1%)	1,27	1,16–1,38	<0,001
Triglicérides (mg/dL)	17 (8,1%)	69 (21,7%)	1,37	1,23–1,51	<0,001
Urina de rotina	41 (19,4%)	104 (49,3%)	1,59	1,37–1,84	<0,001

A função renal, avaliada por níveis de creatinina sérica, foi relatada em 136 (64,5%) pacientes no momento da solicitação. Em relação aos registros por ano, esse parâmetro foi observado em 31 (14,7%) pacientes em 2018, 118 (55,9%) em 2019 e 82 (38,9%) em 2020. Esses dados foram coletados de forma geral, incluindo pacientes que já haviam sido atendidos por nefrologista e outros que ainda aguardavam consulta entre 2018 e 2020. Avaliações da função renal também foram realizadas em 124 (58,8%) e 45 (21,3%) pacientes que estavam sob acompanhamento nefrológico em 2021 e 2022, respectivamente (Tabela 3).

**Tabela 3:** Evolução da taxa de filtração glomerular (tfg), calculada pela equação ckd-epi, do encaminhamento ao nefrologista até 2022

Ano	Pacientes atendidos – N (%)	Média da TFGe (mL/min/1,73 m^2^)	Valor de p	Coeficiente
Encaminhamento	136 (64,45%)	38,64 ± 8,41		
2018	31 (14,69%)	38,95 ± 7,7	0,733	0,57
2019	118 (55,92%)	39,77 ± 11,23	0,217	1,25
2020	82 (38,86%)	37,42 ± 8,8	0,439	–0,88
2021	124 (58,77%)	35,04 ± 8,96	0,001	–3,35
2022	45 (21,33%)	23,48 ± 7,31	0,000	–15,49

Abreviatura – TFGe: Taxa de Filtração Glomerular estimada.

Avaliações de albuminúria, por RAC ou urina de rotina, foram raramente solicitadas, mesmo entre os 76% de pacientes hipertensos e os 49,3% de diabéticos. Embora RAC > 300 mg/g tenha sido observada em alguns pacientes, a maior incidência ocorreu em 2020, com apenas 11 (25%) resultados disponíveis (Figura 2).

**Figura 2:** Níveis de albuminúria medidos pela razão albumina-creatinina urinária (RAC) (mg/g de creatinina) ao encaminhamento e após o acompanhamento nefrológico.

Oitenta por cento dos participantes faziam uso de inibidores do SRAA: 48,3% utilizavam bloqueadores dos receptores de angiotensina e 31,7%, inibidores da enzima conversora de angiotensina (Tabela 1).

A proporção de pacientes que apresentavam resultados laboratoriais disponíveis para cálcio, fósforo, albumina, vitamina D, PTH, ácido úrico, perfil lipídico e sódio—exames recomendados pelas diretrizes para pacientes no estágio 3b—variou de 5% a 32% antes da consulta nefrológica (Tabela 2). Após o atendimento especializado, esses exames foram solicitados e estavam disponíveis para 25–50% dos pacientes, exceto hemoglobina e potássio, que estavam disponíveis para 68% e 58,8%, respectivamente (Tabela 2).

### Complicações Decorrentes da Perda da Função Renal

Os valores médios e a porcentagem de exames fora dos limites ideais definidos pelas diretrizes não diferiram entre os períodos pré e pós-atendimento nefrológico (Tabela 4). Os níveis de potássio sérico estiveram acima de 5 mEq/l em 30% dos pacientes em ambos os grupos (valor máximo de 6,5 mEq/L), embora poucos exames tenham sido solicitados. Anemia, definida como Hb < 12 g/dL, foi observada em 43,6% e 36,1% dos pacientes antes e depois do atendimento nefrológico, respectivamente (Tabela 4). Anemia com indicação terapêutica (Hb < 10 g/dL) foi menos frequente (7,4% e 4,9% nos períodos pré e pós-atendimento, respectivamente).

**Tabela 4:** Resultados de exames laboratoriais dos 211 pacientes com doença renal crônica estágio 3b antes e após a consulta nefrológica

Variáveis	Pré-nefrologista N (%)	Pós-nefrologista N (%)	Valor de P
*Hemoglobina (g/dL)*
Mín–Máx	9,1–17,9	8,4–17,4	
Média (DP)	12,45 ± 1,80	12,82 ± 1,79	
Estratificação
Hemoglobina <10	5 (7,4%)	7 (4,9%)	
Hemoglobina 10–12	26 (38,2%)	45 (31,3%)	0,001^*^
Hemoglobina >12	37 (54,4%)	92 (63,8%)	
Total	68 (100%)	144 (100%)	
Doença Cardiovascular	17 (8%)	32 (15,2%)	
Associação com fatores de risco	0	0	
Hemoglobina <10 cardiovascular			0,5200^*^
Neoplasia	9 (4,3%)	14 (6,6%)	
Hemoglobina <10 neoplasia	1 (0,5%)	1 (0,5%)	
*Albumina (g/dL)*
Mín–Máx	2,9–4,9	2,6–4,8	
Média (DP)	4,02 ± 0,53	4,12 ± 0,42	
Estratificação
Albumina <3,5	1 (9,1%)	4 (5,6%)	0,1430^*^
Albumina >3,5	10 (90,9%)	68 (94,4%)	
Total	11 (100%)	72 (100%)	
*Potássio (mEq/L)*
Mín–Máx	3,5–6,2	3,7–6,5	
Média (DP)	4,76 ± 0,72	4,80 ± 0,51	
Estratificação
Potássio <3,5	0	0	
Potássio 3,5–5	17 (70,8%)	87 (70,7%)	0,2080^*^
Potássio >5	7 (29,2%)	37 (29,8%)	
Total	24 (100%)	124 (100%)	
*Sódio (mEq/L)*
Mín–Máx	136–148	126–145	
Média (DP)	139,96 ± 3,59	139,69 ± 2,95	0,4413^*^
Estratificação
Sódio <135	0	4 (5,6%)	
Sódio 135–145	22 (88,0%)	67 (94,4%)	NA
Sódio >145	3 (12,0%)	0	
Total	25	71 (100%)	
*Cálcio (mg/dL)*			
Mín–Máx	8,2–10,7	4,7–11,6	
Média (DP)	9,37 ± 0,59	9,36 ± 0,75	0,7649^*^
Estratificação			
Cácio <8,5	1 (6,3%)	5 (5,1%)	
Cálcio 8,5–10,5	14 (87,5%)	92 (92,9%)	NA
Cálcio >10,5	1 (6,3%)	2 (2,0%)	
Total	16 (100%)	99 (100%)	
*Fósforo(mg/dL)*			
Mín–Máx	2,9–4,5	2,4–6,6	
Média (DP)	3,66 ± 0,47	3,58 ± 0,71	0,9164^*^
Estratificação			
Fósforo <2,5	0	1 (1,2%)	
Fósforo 2,5–5,6	12 (100%)	80 (97,6%)	NA
Fósforo >5,6	0	1 (1,2%)	
Total	12 (100%)	82 (100%)	
*Vitamina D (ng/mL)*			
Mín–Máx	15–58	8–61	
Média (DP)	30,76 ± 11,31	27,00 ± 11,27	0,6097^*^
Estratificação			
Vitamina D <30	12 (48,0%)	59 (56,7%)	1^*^
Vitamina D >30	13 (52,0%)	45 (43,3%)	
Total	25 (100%)	104 (100%)	
*PTH (pg/mL)*			
Mín–Máx	14–286	12,4–224	
Média (DP)	98,5 ± 96,98	88,92 ± 64,73	
Estratificação 1			
PTH <80	4 (66,7%)	20 (64,5%)	NA
PTH >80	2 (33,3%)	11 (35,5%)	
Total	6 (100%)	31 (100%)	
Estratificação 2			
PTH <150	5 (83,3%)	25 (80,7%)	NA
PTH >150	1 (16,7%)	6 (19,4%)	
Total	6 (100%)	31 (100%)	
*Ácido* ú*rico (mg/dL)*			
Mín–Máx	2–11,8	3,3–12	
Média (DP)	7,3 ± 2,1	7,01 ± 1,62	0,6681^*^
Estratificação
Ácido úrico <6	8 (21,6%)	28 (26,4%)	
Ácido úrico 6–7	4 (10,8%)	20 (18,9%)	0,6010^*^
Ácido úrico >7	25 (67,6%)	58 (54,7%)	
Total	37 (100%)	106 (100%)	
*LDL (mg/dL)*			
Mín–Máx	55–170	44–273	
Média (DP)	92,45 ± 27,01	107,37 ± 43,72	0,6733^*^
Estratificação			
LDL <100	14 (70,0%)	38 (49,4%)	0,4670^*^
LDL >100	6 (30,0%)	39 (50,7%)	
Total	20(100%)	77 (100%)	
*HDL (mg/dL)*			
Mín–Máx	29–73	26–97	
Média (DP)	43,91 ± 12,87	50,23 ± 17,45	0,8527^*^
Estratificação			
HDL <35	4 (36,4%)	10 (8,9%)	NA
HDL >35	7 (63,6%)	43 (81,1%)	
Total	11 (100%)	53 (100%)	
*Triglicérides (mg/dL)*			
Mín–Máx	37–1357	39–2021	
Média (DP)	353,76 ± 391,09	187,01 ± 242,41	0,7532^*^
Estratificação			
Triglicérides <150	5 (29,4%)	36 (52,2%)	NA
Triglicérides >150	12 (70,6%)	33 (47,8%)	
Total	17 (100%)	69 (100%)	
*Urina de rotina*			
Normal	32 (54,4%)	71 (68,3%)	
Glicosúria	1 (2,4%)	3 (2,9%)	
Piúria	1 (2,4%)	1 (1,0%)	
Hematúria	3 (7,3%)	3 (2,9%)	
Albuminúria (+)	2 (4,9%)	19 (18,3%)	
Albuminúria (+ +)	1 (2,4%)	6 (5,8%)	
Albuminúria (+ + +)	1 (2,4%)	1 (1,0%)	
Total	41 (100%)	104	

Abreviaturas – Mín–Máx: mínimo–máximo; NA: não aplicável; PTH: paratormônio; DP: desvio-padrão.

Notas – *Testes de *Wilcoxon* (*signed-rank*) e teste exato de Fisher. Valores de referência do laboratório local para fósforo, conforme recomendado pela KDIGO.

### Redução da Função Renal

O modelo linear misto demonstrou que não houve declínio significativo da função renal, medido por TFGe, durante o período pré-atendimento com o nefrologista. Entretanto, houve declínio da função renal após o início do acompanhamento nefrológico, com reduções em 4 (3,25 mL/min) e em 5 anos (15,49 mL/min) após o encaminhamento (Tabela 3).

## Discussão

Nossos resultados indicam que os médicos generalistas aderem com pouca frequência às diretrizes da KDIGO para o encaminhamento de pacientes a um nefrologista ou para o acompanhamento adequado das avaliações laboratoriais indicadas. No entanto, tais profissionais desempenham um papel importante no manejo abrangente da doença renal crônica. Sua contribuição vai além da detecção precoce e do monitoramento da insuficiência renal, abrangendo a prevenção de hospitalizações e a coordenação do cuidado multidisciplinar. Estudos anteriores demonstraram que a participação do médico generalista pode reduzir o risco de hospitalização, mesmo durante períodos desafiadores, como a pandemia de COVID-19^26,27,28^.

O tempo médio até a primeira consulta com o nefrologista foi de 20 meses, e a maioria dos pacientes foi atendida duas a três vezes por ano, em conformidade com as diretrizes, tanto antes quanto depois do acompanhamento com nefrologista. Notavelmente, não houve diferenças entre o pré e o pós-acompanhamento nefrológico na frequência de complicações da DRC, como anemia, hipercalemia e hipocalcemia. Entretanto, observou-se um declínio na TFGe ao comparar os dois últimos anos com os dados basais.

Em pacientes com DRC, a perda esperada de TFGe é relatada entre 0,3 e 1 mL/min/ano^8,24,29,30^, o que está em consonância com nossos achados nos primeiros três anos de acompanhamento. No entanto, observamos um declínio acelerado da função renal nos últimos quatro e cinco anos, excedendo o limiar de 5 mL/min/ano sugerido pela KDIGO. Permanece incerto se o declínio observado da função renal esteve relacionado à menor frequência de consultas médicas de rotina, ao acesso limitado a exames laboratoriais ou aos efeitos diretos ou indiretos da infecção pelo SARS-CoV-2. Contudo, dado o delineamento retrospectivo deste estudo, não foi possível controlar potenciais fatores de confusão nem estabelecer uma relação causal entre a pandemia e o declínio da função renal observado. Essa limitação deve ser reconhecida e considerada na interpretação desses achados^1,31^.

O estudo *Chronic Kidney Disease Outcomes and Practice Patterns Study* (CKDopps)^32^ avaliou pacientes em pré-diálise sob cuidados nefrológicos em vários países, incluindo 888 pacientes do Brasil; no entanto, apenas 31 estavam no estágio 3b. Nosso estudo, em contraste, focou em pacientes inicialmente manejados na atenção primária, destacando lacunas atuais no cuidado não especializado dessa população. Notavelmente, a coorte do CKDopps tinha 10 anos a menos que a população de nosso estudo, mesmo considerando apenas pacientes com DRC estágio 3b.

Observamos uma preocupante falta de disponibilidade de resultados laboratoriais para pacientes com DRC acompanhados por médicos generalistas ou nefrologistas. Menos de 25% dos exames recomendados pelas diretrizes estavam disponíveis em nosso estudo, semelhante ao relatado pelo CKDopps^32^, onde os resultados de albuminúria estavam disponíveis para menos da metade da população estudada. Não está claro se isso se deve ao desconhecimento dos médicos sobre as diretrizes ou à falha dos pacientes em realizar os exames solicitados.

Comorbidades como diabetes e hipertensão são prevalentes nessa população e exigem controle e acompanhamento adequados^33,34^. Mesmo o exame de RAC, de rotina, esteve raramente disponível para os participantes do estudo. A prevalência de hipertensão em pacientes com DRC estágio 3b foi consistente com relatos anteriores^33,35^. A anemia, definida como Hb < 12 g/dL, também foi prevalente, apesar do acompanhamento nefrológico. No entanto, no Brasil, o tratamento da anemia em pacientes com DRC, que inclui eritropoetina e suplementação de ferro, é fornecido pelo SUS apenas quando o nível de Hb é inferior a 10 g/dL^36^, representando menos de 10% antes e após o acompanhamento nefrológico.

Este estudo observacional é o primeiro a acompanhar pacientes com DRC estágio 3b manejados na atenção primária. Incluímos todos os pacientes elegíveis que estavam na lista de espera no momento do estudo. Além disso, o sistema integrado do SUS oferece uma oportunidade única para compreender múltiplos aspectos do cuidado a esses pacientes dentro da rede pública de saúde (por exemplo, acesso a consultas e resultados de exames laboratoriais). No entanto, por se tratar de um estudo retrospectivo, a qualidade dos dados extraídos dos prontuários eletrônicos pode ter impactado a consistência das análises e a robustez das conclusões. A disponibilidade limitada de dados laboratoriais evidencia um desafio relevante para garantir o acompanhamento adequado dos pacientes. Permanece incerto se esses exames não foram solicitados pelos profissionais de saúde ou se não foram realizados pelos pacientes, o que merece investigação adicional. Além disso, por utilizar uma amostra de conveniência restrita a uma única cidade, os achados podem não ser generalizáveis para outras regiões brasileiras que apresentam diferenças na estrutura do sistema de saúde, na distribuição de recursos e nas características populacionais. Apesar dessas limitações, o estudo retrata de forma fidedigna o manejo da DRC na prática real da atenção primária e enfatiza barreiras persistentes relacionadas à adesão às diretrizes clínicas e à coordenação entre os diferentes níveis de atenção. Os resultados contribuem para identificar lacunas que podem orientar estratégias voltadas ao aprimoramento da prática clínica e da gestão do cuidado para pacientes com DRC.

## Conclusão

Embora os pacientes com DRC estágio 3b fossem atendidos com frequência por seus médicos, a ausência de exames laboratoriais conforme recomendado pela KDIGO levanta preocupações quanto à adequação do cuidado prestado. Por outro lado, não foi observado declínio significativo da função renal nos primeiros dois anos, período que correspondeu à mediana de tempo até a consulta com o nefrologista. Entretanto, o monitoramento insuficiente de complicações essenciais, como albuminúria, anemia e distúrbios minerais e ósseos, indica que a ausência de queda na TFGe, isoladamente, não pode ser interpretada como evidência de segurança clínica durante esse período de espera. Investigações futuras devem avaliar o impacto do atraso na consulta nefrológica sobre a progressão da DRC e suas complicações no âmbito da atenção primária, particularmente no contexto do apoio matricial em nefrologia. Além disso, esforços devem ser direcionados ao aprimoramento da implementação das diretrizes clínicas e à compreensão das barreiras à sua adesão. Esses achados ressaltam a necessidade de educação profissional contínua e de um apoio matricial estruturado no SUS, visando elevar a qualidade do cuidado destinado aos pacientes com DRC estágio 3b.

## Data Availability

Os dados estarão disponíveis mediante solicitação.
